# Discovery of CNS-Like D3R-Selective Antagonists Using 3D Pharmacophore Guided Virtual Screening

**DOI:** 10.3390/molecules23102452

**Published:** 2018-09-25

**Authors:** June Hyeong Lee, Sung Jin Cho, Mi-hyun Kim

**Affiliations:** 1 Gachon Institute of Pharmaceutical Science & Department of Pharmacy, College of Pharmacy, Gachon University, 191 Hambakmoeiro, Yeonsu-gu, Incheon 21936, Korea; ljhyung1017@naver.com; 2 CimplSoft, Thousand Oaks, CA 91320, USA; sjcho@cimplsoft.com; 3 CimplRx, Euni-ro, Seoul 06764, Korea

**Keywords:** pharmacophore, 3D-QSAR, virtual screening, D3R selective antagonist, molecular docking, CNS-like

## Abstract

The dopamine D3 receptor is an important CNS target for the treatment of a variety of neurological diseases. Selective dopamine D3 receptor antagonists modulate the improvement of psychostimulant addiction and relapse. In this study, five and six featured pharmacophore models of D3R antagonists were generated and evaluated with the post-hoc score combining two survival scores of active and inactive. Among the Top 10 models, APRRR215 and AHPRRR104 were chosen based on the coefficient of determination (APRRR215: R^2^_training_ = 0.80; AHPRRR104: R^2^_training_ = 0.82) and predictability (APRRR215: Q^2^_test_ = 0.73, R^2^_predictive_ = 0.82; AHPRRR104: Q^2^_test_ = 0.86, R^2^_predictive_ = 0.74) of their 3D-quantitative structure–activity relationship models. Pharmacophore-based virtual screening of a large compound library from eMolecules (>3 million compounds) using two optimal models expedited the search process by a 100-fold speed increase compared to the docking-based screening (HTVS scoring function in Glide) and identified a series of hit compounds having promising novel scaffolds. After the screening, docking scores, as an adjuvant predictor, were added to two fitness scores (from the pharmacophore models) and predicted Ki (from PLSs of the QSAR models) to improve accuracy. Final selection of the most promising hit compounds were also evaluated for CNS-like properties as well as expected D3R antagonism.

## 1. Introduction

Dopamine receptors are a class of G protein-coupled receptors in the central nervous system (CNS). There are at least five subtypes of dopamine receptors: D1R, D2R, D3R, D4R and D5R. The D1R and D5R are members of the D1-like family of dopamine receptors, whereas the D2R, D3R, and D4R are members of the D2-like family [1]. The D3R is found within a key neuronal network involved in motivation and cognition. In contrast to the D2R, the D3R does not appear to play a role in the regulation of movements [2,3]. Furthermore, the D3R subtype is considered to represent an important target for the treatment of a variety of neurological diseases, such as schizophrenia and Parkinson’s disease [4]. D3R-selective antagonists can be potential therapeutic agents for the treatment of psychostimulant addiction and relapse [5,6,7,8,9]. However, many clinically approved drugs targeting the D3R do not show significant selectivity over the D2R and other receptors [9]. Thus, it is necessary to develop selective D3R antagonists.

**Figure 1:**
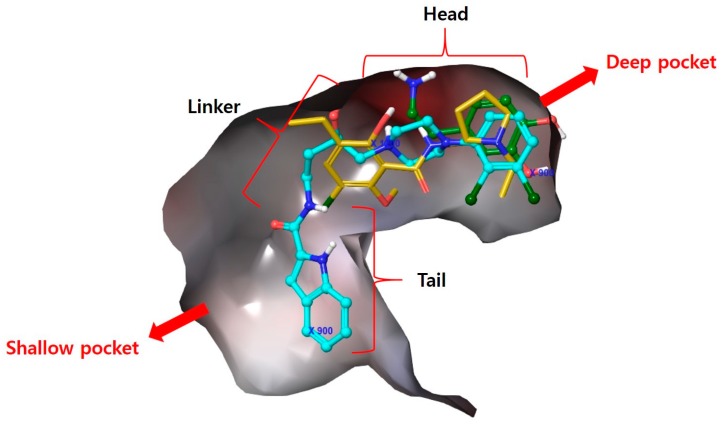
Superimposed 3PBL protein-ligand docking of dopamine (green; endogenous D3R agonist), eticlopride (yellow; non-selective D3R antagonist) and R-22 (sky blue; selective D3R antagonist).

In this study, we have conducted computer-aided drug identification to investigate D3R-selective antagonists for use in CNS diseases. After examining structural differences between the D3R (PDB name: 3PBL) and the D2R proteins [10], binding modes of three ligands (dopamine, eticlopride, and R-22) with D3R were comparatively analyzed using docking simulations: (1) dopamine (as the endogenous ligand); (2) eticlopride (as a non-selective D2R/D3R antagonist [11]); and (3) R-22 (as a selective D3R antagonist). According to the literature [12], our docking approach identified a common binding site, a deep pocket common to all three compounds, and a shallow pocket occupied by some antagonists (Figure 1). R-22, a selective D3R antagonist, occupied the deep pocket and was found bound to the outer binding pocket to a greater extent [12]. The residue information derived from the docking study was used to develop novel molecules [13], and models including pharmacophore features were built from the dataset containing selected D3R antagonists with high efficacy, selectivity, and metabolic stability.

Rational drug design is based on the notion that molecules are active at a particular receptor because they have numerous chemical features and complementary geometries that facilitate their interaction with the binding site [14]. These pharmacophore features reflect the atoms present, their locations, and six key representation of non-covalent interactions: Hydrogen bond acceptor, hydrogen bond donor, hydrophobicity, positive & negative ionizable moieties, and aromatic ring. A pharmacophore hypothesis is a collection of 3D vectors which represent these features. To discover pharmacophores in the absence of reliable co-crystallographic data, the active analog approach is typically used to facilitate the identification of common protein-ligand interactions [15]. In addition, pharmacophore model can compensate insufficient docking prediction resulting from a large deviation between docking pose and co-crystallographic data in some target like D3R. Herein, we tried to build pharmacophore model able to supplement D3R docking models as well as to describe the models.

## 2. Materials and Methods

### 2.1. Definition and Whole Workflow of the Study

**Figure 2:**
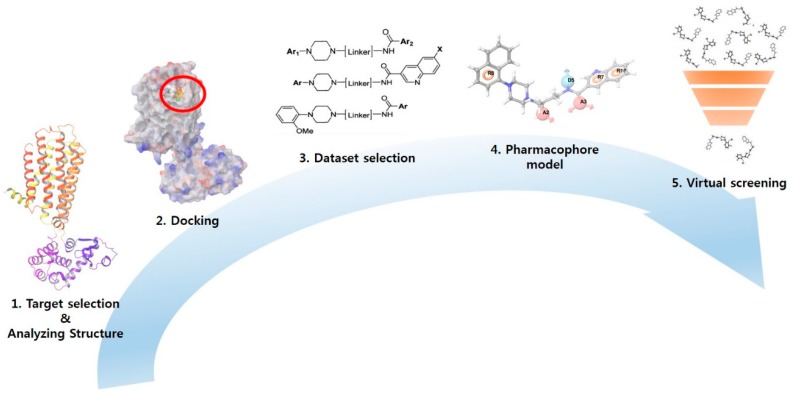
Workflows of this study.

To build reliable predictive models for D3R-antagonists, a user must set a minimum number of features for building a pharmacophore model without overfitting. The common pharmacophore generation is conducted to identify top-ranked active compounds. Scoring is based on the vector score, site score, volume score, selectivity score, and clustering. The scoring of active compounds and overall ranking of plausible models may not be enough to select more reliable models describing the binding mode of active compounds. By including the scoring of inactive compounds, pharmacophore models were compared. From chosen pharmacophore models, 3D-QSAR models could be developed using the conformers of compounds in the training set, which match the pharmacophore on sites. The employed fitness indicates an alignment tendency of each molecule. After processing, generating, and validating models, two optimal D3R-antagonist models were used to screen compounds obtained from commercial databases. After considering how to make robust models, the question of how to use the models was depicted in the workflow (Figure 2).

### 2.2. Data Collection and Molecular Docking

A set of 25 novel D3R-selective antagonists (Table 1) [16] and their inhibition constant (Ki) data (nM) were utilized for the development of a pharmacophore model and building pharmacophore-based three-dimensional quantitative structure–activity relationship (3D-QSAR) model [16]. The negative logarithms of the measured Ki values (pKi for human dopamine D3R binding data in human embryonic kidney cells) were converted to μM, which was used in this study. Before building a pharmacophore model, compounds were processed by Ligprep to attach hydrogens, convert 2D structures to 3D, and generate stereoisomers [17]. In addition, we applied the target pH of 7 ± 2 using Epik, and specified chirality using Force field OPLS_2005 in Maestro 10.4, Schrödinger [18]. After ligand preparation, each ligand had five conformers and every conformer was used for docking to D3R. For the docking, various docking models between R-22 and the apo protein of 3PBL were generated using (1) residue specific centroid selection; (2) adjustment of grid size & shape; (3) constraint on the residues having expectable non-covalent interaction; and (4) three scoring functions. The optimal model was selected based on the docking score and similarity of the pose compared to one found in the literature [12]. The best docking condition of R-22 was used to dock 125 conformers to generate the best pose of each conformer.

### 2.3. Identification of Pharmacophore Hypotheses

Pharmacophore models were built using Pharmacophore Alignment and Scoring Engine (PHASE) running on Maestro 10.4 (Schrödinger). The best docking pose was considered to be a bioactive conformer of each conformer and used for the development of the pharmacophore model. The activities of compounds were scaled from a minimum value of 1.01 to a maximum value of 3.93 (to be consistent with ones in the table), with an activity threshold of 1.35; this meant that compounds with a Ki of <44 nM were considered to be antagonists (actives). The pharmacophore features used for hypothesis generation were hydrogen bond acceptor (A), hydrogen bond donor (D), hydrophobic group (H), positively ionisable (P), negatively ionisable (N), and aromatic rings (R) defined by a set of chemical structure patterns. For the current dataset of 125 conformers, five or six features were chosen for model construction. The pharmacophore feature of active ligands that contain identical sets of features with very similar spatial arrangements were grouped together to give rise to a common pharmacophore hypothesis. In the present study, pharmacophore-based QSAR modeling was conducted by dividing the dataset into a 35-member training set (70%) and a 15-member test set (30%) in a random manner. The chemical features associated with ligand-protein binding and the training set data are used to create a model, and the test set is used to evaluate this model [19].

### 2.4. Internal and External QSAR Model Validation

The success of a virtual screening campaign using a QSAR model depends heavily on the quality of the model. Several statistical parameters in PHASE, such as R2 for the training set and Q2 for the test set, the standard deviation, root mean square error, and variance ratio (F), can be used to evaluate the robustness of a QSAR model [20,21,22,23]. To test the reliability of QSAR models, compounds that were not used for model development must be used: (1) test set (15 compounds); (2) 3rd set (out of initial dataset). The external validation of the 3rd set was conducted using D3R antagonists extracted from the ChEMBL database. ChEMBL compounds with D3R Ki value were imported and filtered using the active cut-off of pKi > 1.5 uM and the inactive cut-off of pKi < 1.0 uM. The selected compounds were then docked to 3PBL. This external validation was followed by pharmacophore based screening with QSAR prediction in the condition of (1) generation of conformer ensembles within the energy window (20 Kcal/mol); (2) minimum pharmacophore feature matching of 4 out of 5, 2 out of 5, 2 out of 6 or 5 out of 6 site points. The predictive values derived from this screening process were then compared with the experimental values.

The pharmacophore models were further validated by a receiver operator characteristic curve (ROC) analysis. Therefore, it was necessary to prepare a test set that included active and inactive compounds. The imported 3rd set for pharmacophore-based prediction was processed on the basis that the pKi values of active compounds (n = 1111) were >1.5, the pKi values of inactive compounds (n = 1416) were <1.0, molecular weight was between 400 and 500, ALogP was between −2 and 6, the number of hydrogen bond donors is <4, and the number of hydrogen bond acceptors is <8. The ROC curve analysis describes the sensitivity (true positive rate, Se) for any possible change in the number of selected compounds as 1–Sp (specificity, which is defined as the true negative rate) [24].

$$\;\mathrm{Se}=\frac{number\;of\;selected\;actives}{total\;number\;of\;actives}=\frac{TP}{TP+FN}\;$$

$$\;\mathrm{Sp}=\frac{number\;of\;discarded\;inactives}{total\;number\;of\;inactives}=\frac{TN}{TN+FP}\;$$

In the ROC analysis, true positive (*TP*) means that the active compound is measured as active in the test. Likewise, true negative (*TN*) means to infer that inactive compound is measured as inactive. In contrast, false positive (*FP*) means that inactive is measured to active and false negative (*FN*) means that active is measured to inactive. A model with fewer *FP* and *FN* errors is a superior model, and selectivity and specificity are the ratios of these. R software (version 3.3.2) was used to plot ROC curves, calculate the area under the curve (AUC), and generate box plots, to compare the range of experimental and predictive values.

### 2.5. Pharmacophore-Based Virtual Screening 

The validated pharmacophore models were chosen for queries in our pharmacophore-based virtual screening [15]. Screening was conducted to identify hit compounds with chemical features corresponding to those of the template [25]. If a molecule can be fitted inside pharmacophore features, it could be considered a hit molecule based on the fitness score [26]. Existing conformers of all ligands in the database were included and screened for matches on at least 4 out of 5 or 5 out of 6 site points using the advanced pharmacophore method in PHASE (Schrödinger) running on a Linux-x86_64. The output of this QSAR model represented a maximum of 100,000 hits (≤1 hit per molecule) and considered atom types when computing volume scores. The commercially available compound library from eMolecules was used as the source of screening compounds to find novel hit compounds.

### 2.6. Hit Selection

The screened in silico hits were docked to the D3R using the best docking condition identified when R22 was docked to the apo protein (3PBL). The processed compounds were then subjected to filtering, and the selected hit molecules were grouped by *k*-means clustering using binary fingerprints generated with MACCs in Canvas. According to general optimal *k* decision rules: (1) maximum betweenness of clusters as separation of clusters; (2) minimum withinness in a cluster as cluster tightness or homogeneity in explicit clustering, fingerprint based *k*-means clustering proposed us ten as the optimal *k* so that they were divided into 10 clusters based on their molecular similarity. Representative compounds from these clusters were then selected after considering their estimated efficacy, docking scores, and CNS drug-like properties. 

A summary of the hit selection workflow is summarized in Figure 2. Hit compounds identified by screening using two models were sorted by fitness score in descending order; then, they were filtered using the following drug-like criteria based on Lipinski’s rule: AlogP < 5; molecular refractivity of 4~130; nitrogen-containing; Molecular weight (MW) of 180~500 and preferably around 400; Polar surface area (PSA) < 90; Hydrogen bonding donor (HBD) < 5; and Hydrogen bonding acceptor (HBA) < 10 [27]. Filtered hits were processed through Ligprep for conversion to 3D, docked to 3PBL to investigate their interaction with the target protein, and filtered using the secondary criteria of a docking score < −7, a ligand efficiency < −0.3 [28], and a charge range from −1 to 1. Qikprop in Maestro is used to apply CNS-like property filtering criteria of QPlogBB > −0.523, BB > 0.3 [29,30,31], and CNS ≥ 1; the score value of −2 is CNS inactive, and the score value of 2 is CNS active [32]. 

### 2.7. Cell-Based β-Arrestin Assay of D3R and D2R

β-Arrestin assay was conducted through non-imaging assay monitoring the activation of D2R/D3R using a technology developed by DiscoverX. A specific peptide tagged β-glactosidase (β-Gal) as a functional reporter was fused with tested D2R or D3R and an enzyme acceptor (EA) of the β-Gal was fused with β-arrestin. The fused proteins were overexpressed in the CHO-K1 cell line. In the cell line, when D2R or D3R is activated and β-Arrestin is recruited to the GPCRs, the peptide tag of β-Gal and EA complementation happens, restoring β-Gal activity which is measured using PathHunter, chemiluminescent detection reagents containing substrate of β-Gal with a PerkinElmer Envision^TM^ instrument. For measuring the antagonism of risperidone, a positive control and 9 compounds, the cultured cells were pre-incubated with the test compounds (conc. 10 μM) followed by agonist at the EC80 concentration (conc. 0.072 μM). Intermediate dilution of sample stocks was performed to generate 5-fold sample in assay buffer. The sample was added to cells and incubated at 37 °C or room temperature for 0.5 h. 6-Fold diluted EC80 agonist (dopamine) in assay buffer was added to the cells and incubated at 37 °C or room temperature for 1.5 h. Assay signal was generated through a single addition of 12.5 or 15 μL (50% *v*/*v*) of the detection reagent cocktail, followed by 1 h incubation at room temperature. The percentage inhibition was calculated using the following formula using CBIS data analysis suite:

% Inhibition = 100% × (1 − (mean RLU of test sample − mean RLU of vehicle control)/(mean RLU of EC80 control − mean RLU of vehicle control))

## 3. Results and Discussion

### 3.1. Docking Analysis of D3R Selective Antagonists

**Figure 3:**
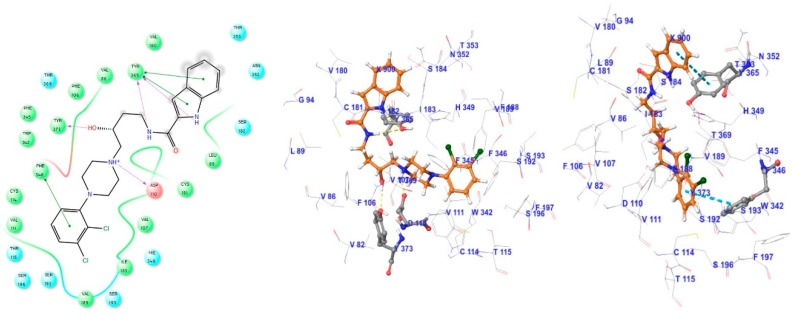
Docking pose showing the interaction between R-22 and 3PBL residues. In the left-hand picture, the blue line shows Pi-Pi stacking, the purple line shows hydrogen bond interaction and the yellow-green line shows hydrophobic interaction.

The high degree of sequence identity within the transmembrane helixes of the D2R and D3R (78%) implies that these receptors have similar binding sites [33]. Due to this reason, it has been difficult to develop D3R-selective antagonists with drug-like physicochemical properties [34,35]. To clarify the ligand-protein binding features of the D3R, we performed ligand docking to 3PBL using dopamine, eticlopride (D2R/D3R dual antagonist) [11] and the D3R-selective antagonist, R-22 under the various condition including (1) the center, size, and shape of grid box; (2) constraints on non-covalent interactions with reported key residues (e.g., Glu90, Asp110, Ser192, Ser193, Phe197, Tyr373 and His349) and (3) three scoring functions. As the result, the proposed binding mode of R-22 in the literature [12] was reproducible under the optimal docking condition showing the non-covalent interactions with important residues included Asp110, Tyr365, Tyr373, and Phe346 (Figure 3). From the docking condition, docked dataset compounds also showed the occupation of the outer shallow pocket as well as the deep pocket where the endogenous agonist binds to. Based on the binding mode, it is reasonable to develop selective D3R antagonists that target the shallow pocket. Conversely, the deep pocket is a common binding region where agonists and antagonists interact. It is a common expectation that docking within the deep pocket is necessary for targeting this protein, and the pharmacophore should therefore interact with both the shallow and deep pockets. In addition, ligands including the hydroxy group in the linker showed a reduced potency.

### 3.2. Pharmacophore Modeling and 3D-QSAR Models

The knowledge from docking analysis made us consider models containing five or six features to describe the interactions with two pockets. After building models to explore every plausible hypotheses from training set, the next scoring technique was applied to evaluate the models according to site, vector, volume, selectivity, conformational energy and activity.

Survival score = *W*_site_·*S*_site_ + *W*_vec_·*S*_vec_ + *W*_vol_·*S*_vol_ + *W*_sel_·*S*_sel_ + *W^m^*_rew_ − *W*_E_·Δ*E* + *W*_act_·*A*

**Figure 4:**
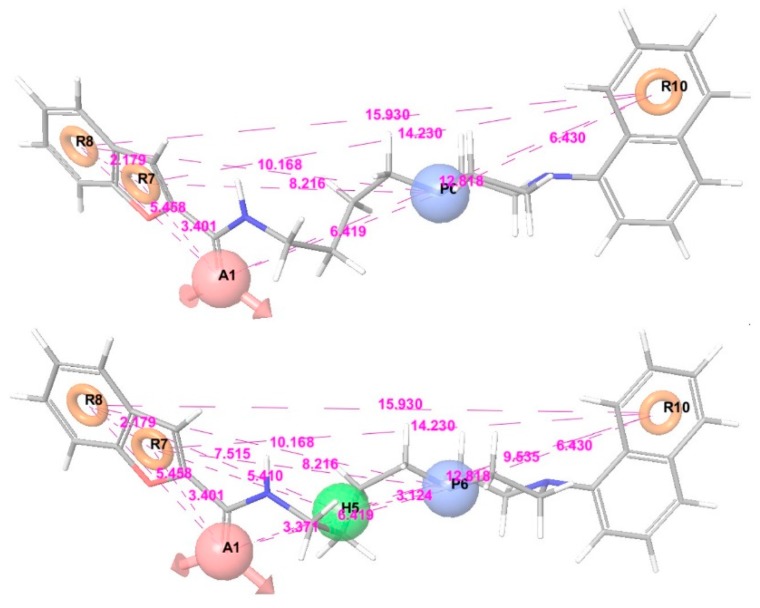
Representative 3D-QSAR models: APRRR215 (upper) and AHPRRR104 (lower).

**Figure 5:**
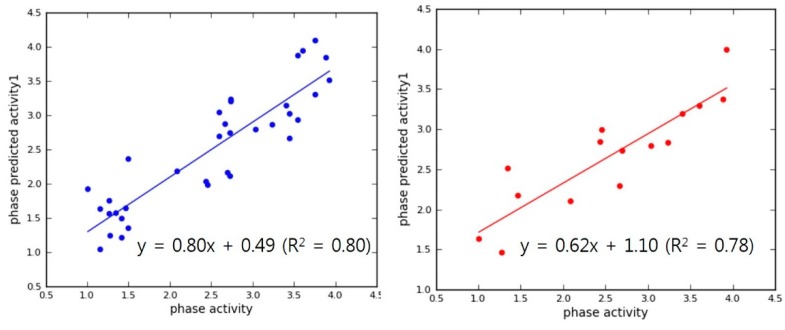
APRRR215 regression lines for the training (blue) and test (red) sets.

**Figure 6:**
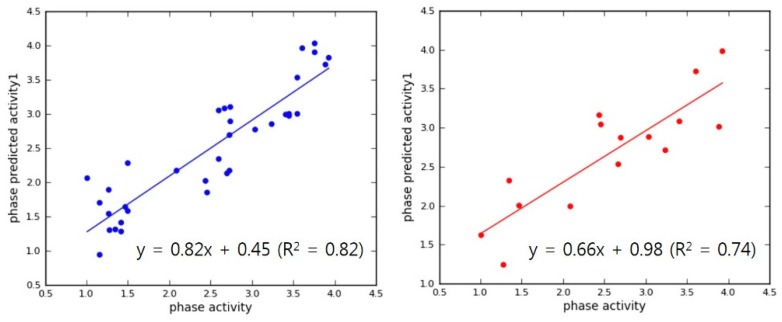
AHPRRR104 regression lines for the training (blue) and test (red) sets.

The selection of superior hypothesis was committed based on an outstanding post-hoc survival score combining two survival scores of active and inactive. Top 10 pharmacophore models showing superior post-hoc scores were used to generate 3D-QSAR models. Internal validation of the chosen PLS models produced reliable coefficient of determination (R squared of each hypothesis >0.7, Max. 0.88) and their high calibrated values (Q squared of each hypothesis >0.6, Max. 0.73) in Table 2. In addition, Pearson correlation constants also showed more than 0.8 in all of our chosen models. Finally, two models (APRRR215 and AHPRRR104) were selected for virtual screening based on these statistical analyses and the appropriate location of features (Figure 4). In the simple regression analysis of the experimental activities with predicted activities, the best model, APRRR215, presented Act_(pred.)_ = 0.80·Act_(exp.)_ + 0.49 (R_predictive_ squared = 0.80) in the training set and Act_(pred.)_ = 0.62·Act_(exp.)_ + 1.10 (R_predictive_ squared = 0.78) in the test set (Figure 5). Another chosen model, AHPRRR104, indicated that Act_(pred.)_ = 0.82·Act_(exp.)_ + 0.45 (R_predictive_ squared = 0.82) in the training set and Act_(pred.)_ = 0.66·Act_(exp.)_ + 0.98 (R_predictive_ squared = 0.74) in the test set (Figure 6).

**Figure 7:**
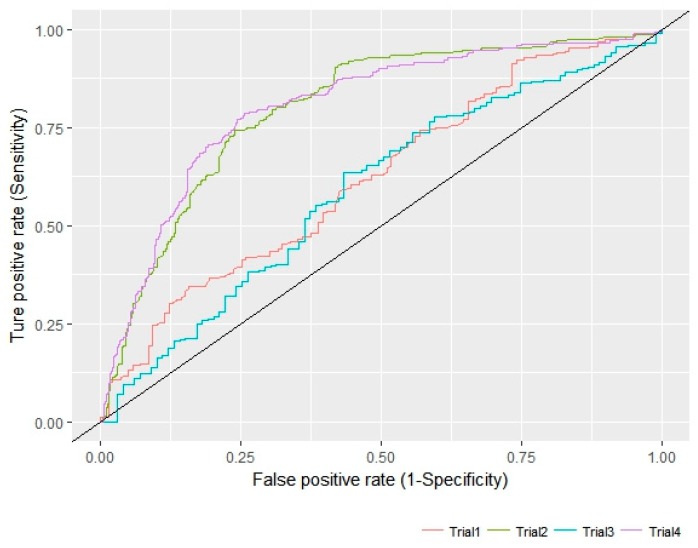
APRRR215 ROC curve and AUC.

**Figure 8:**
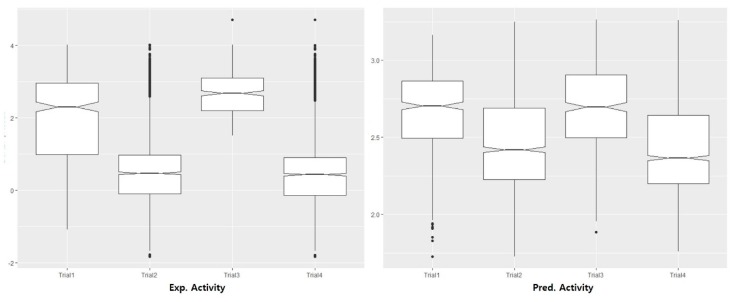
APRRR 215 skeletal box-and-whisker plot.

The external validation of the best model, APRRR215 was further conducted using 3rd data set (known D3 antagonists with structural diversity) from the ChEMBL database. Visualization of the validation was presented in ROC analysis to show how effectively the pharmacophore models distinguished between active and inactive compounds. Sensitivity (in other words, true positive rate, recall, hit rate) and specificity (in other words, true negative rate) are general indices to show the predictive power of a validated model. The ROC curve is a graphical representation of the false positive rate, (1–specificity) of *x*-axis and the true positive rate, sensitivity of *y*-axis for each of the possible cut-offs [36]. The accuracy of this test is measured by the AUC of the ROC curve. According to the general judgement on an AUC value of ROC curve (AUC of poor model = 0.5, AUC of a moderate accurate model = 0.5 to 0.7, and very accurate if the AUC > 0.9) [37], the AUC of two models in four trials could show moderate usefulness in global scaffolds: AUC of Trial 1 = 0.623 (≥4 out of 5 in APRRR215), AUC of Trial 2 = 0.801 (≥2 out of 5 in APRRR215), AUC of Trial 3 = 0.592 (≥5 out of 6 in AHPRRR104), and AUC of Trial 4 = 0.805 (≥2 out of 6 in AHPRRR104) (Figure 7). The constraint of pharmacophore feature matching in pharmacophore-based QSAR prediction made an effect on model performance so that it showed that our models are more sensitive to recognizing mismatched inactive but have the limited performance in delicate recognition of inactive having similar pharmacophore. In addition, the distribution of the experimental and predictive values was visually compared through box-and-whisker plot in Figure 8. Even though the model presented the limit to residual between experimental value and predicted value of active compounds, median values of two distributions could be well-matched and two distributions were identical after normalization. 

### 3.3. Identification of CNS-Like D3R Antagonist through Virtual Screening

**Figure 9:**
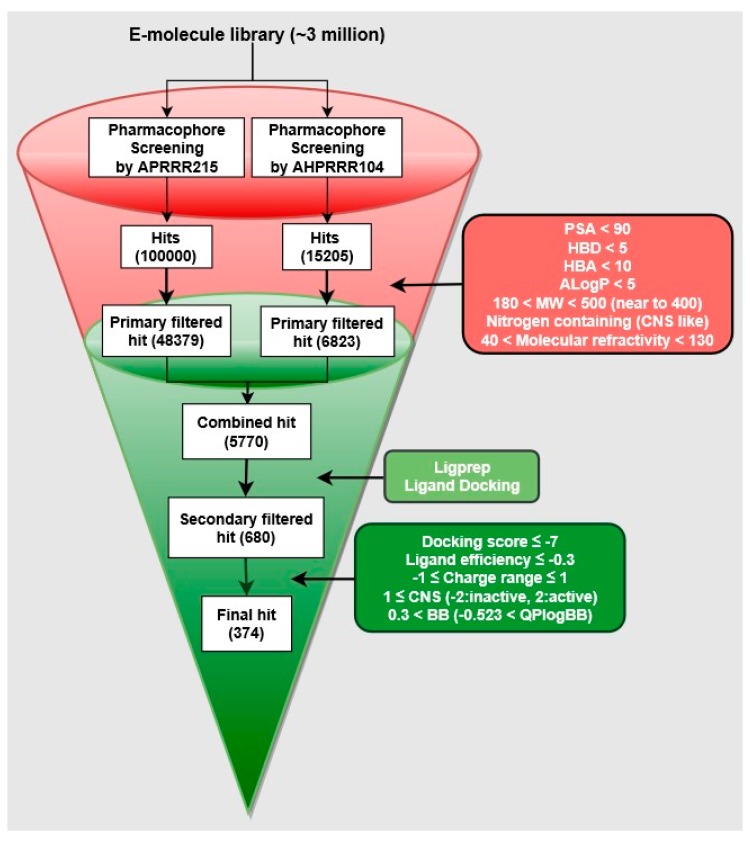
Hit selection workflow.

The two reasonable pharmacophore hypotheses (APRRR215 and AHPRRR104) showing best statistics in their 3D-QSAR were used as templates for virtual screening [19]. The screening identified in silico hit compounds with chemical features corresponding to those of the template [19,23,26]. Some of these hits might be similar to known active compounds, while others might have more novel scaffolds [23,38]. The identification of hit compounds having different scaffolds was the primary aim of our research, development of novel D3R-selective antagonists. Our pharmacophore-based virtual screening played the role of high-throughput filter against compounds that lack the features essential for binding. The validated two best pharmacophore models allowed a researcher to efficiently screen a large commercial compound library from eMolecule (more than 3 million), and the results were filtered to identify compounds with CNS drug-like properties (Figure 9).

APRRR215 and AHPRRR104 resulted in 1,000,000 and 15,205 hits, respectively, and these were filtered by drug-like criteria, MW, AlogP, HBD, HBA, molecular refractivity, nitrogen-containing and PSA. In particular, PSA is commonly used to indicate whether a CNS drug can penetrate the cell membrane through passive diffusion. The PSA of a CNS drug is required to be <90 angstroms to penetrate the blood brain barrier [39]. The numbers of filtered hit molecules were 48,379 for APRRR215 and 6823 for AHPRRR104, with 5770 compounds in common. Even though drug property filtering had already been performed in the pharmacophore hit selection steps, the CNS drug-like properties were reconsidered for the 3D conformation and filtered. In addition, binding poses and docking scores of 5770 compounds with D3R (apo protein of PDB: 3PBL) were considered to present the most selective D3R antagonists interacting with both the deep and shallow pockets. Consequently, 374 compounds were extracted as filtered hits. The primary aim of this study was to find compounds with different scaffolds from the known D3R antagonists (a template), sharing the pharmacophore features. We identified 10 (a representative member of each cluster) novel hits predicted to act as dopamine D3R antagonists with appropriate estimated efficacies and pharmacokinetic properties (Table 3). The final selection also was experimentally validated by efficiency of the in silico D3R antagonists, of which cell-based β-arrestin assay (PathHunter^®^) was measured in D3R overexpressed cell line. In addition, D3R selectivity was checked through the comparison with D2R antagonism in the same assay condition except for dopamine concentration due to Ki ratio of dopamine against D2R/D3R = 489. When the percent efficacy of tested compounds is calculated by (raw value − EC80 of dopamine)/EC80 of dopamine × 100. While the Ki ratio range of risperidone (a positive control compound) was from 0.1 to 0.8 in ChEMBL database, the concentration ratio of D2R/D3R for showing the same % efficacy was 0.1 in the β-arrestin assay so that % efficacy ratio of test molecules between D3R and D2R can be approximate surrogate value of real D2R/D3R Ki ratio of them. In the spite of intensive structure tuning of linker part and tail part, the entry 9 presented better efficacy than other entries but could not made us expect high D2/D3 selectivity. The entry 1 presented the highest selectivity among all entries and relatively better efficacy but could not show enough structural novelty discriminative with known D3R antagonists. Despite scaffold similarity with training set, the entry 5 could not show enough antagonism as well as D2/D3 ratio. Remaining entries having structural novelty could not show promising selectivity or efficacy. Despite the limited result, the entry 1 and entry 9 enabled us to retrieve new models and to further study SAR of the two scaffolds due to selectivity control and activity.

## 4. The Current Limitation of This Study & Future Possibilities

The external validation result of APRRR215 indicated that the QSAR model could show moderate predictive power with discriminating ability in binominal decision but could not predict pKi value on D3R with high accuracy. Plausible reasons for the large deviation between experiment and prediction in 3rd set are the following: (1) current models built from the long-shape drug scaffold can permit narrow deviation of a drug shape for the prediction; (2) D2R/D3R-selectivity ratio was not considered in our model (ex. D2R/D3R ratio > 10 in active) but two variables, Ki value and the selectivity, can’t be independent and the selectivity as a confounding factor perfectly can’t be controlled in our current 3D-model; (3) flexibility of 3D-coformation, in particular, the flexibility of D3R antagonists in shallow pocket can make quantitative prediction difficult. In the future, plausible trials and advances from the trials are here: (1) more than two dependent variables (activity, D2/D3-selectivity, and so on); (2) extension of dataset; and (3) well-designed conformational sampling of data set for building an advanced model [40].

Despite the limitation of current models, we can consider the advantages and contribution of the models. Firstly, our pharmacophore model can effectively contribute to finding novel compounds with high 3D similarity but low 2D similarity. In general, when designing a new scaffold from a known template molecule, medicinal chemists tend to consider 2D similarity than 3D. However, if a drug is 2D-dissimilar to existing drugs for the cognate target but has high 3D similarity, there is a greater likelihood of obtaining a novel pharmacological effect [38,41,42]. In addition, the measured distances between the pharmacophores are consistent with the proposed binding conformer of R-22 and each pharmacophore feature could describe non-covalent bonding interaction between R-22 and corresponding residues. Secondly, in the view of screening efficiency, the pharmacophore-based screening was 100-fold faster than high-throughput docking without any pre-processing in one sub job. Under the condition of Intel Xeon E5-2650v2 (20 M Cache, 2.60 GHz, 8.00 GT/s, 8 × 4 cores), HTVS docking of 100,000 chemicals from eMolecules (>3 million compounds) consumed total 57,959 seconds (16 h) CPU time in 16 parallel sub jobs. Under the same resource, the pharmacophore-based screening of whole eMolecule chemicals could be completed within 72 h in one job. Because parallel (thread) processing of both screenings does not have dependency between sub jobs, the pharmacophore-based screening was also superior to docking in the speed of total job. Finally, double filtering through two screening and data fusion of scored values (e.g., the fitness score of pharmacophore model, predicted pKi of QSAR, and docking score) can improve predictive power [43,44].

## 5. Conclusions

In this study, we have developed pharmacophore models of D3R selective antagonists to efficiently identify a series of compounds with novel scaffolds, having both predicted D3R antagonism and CNS drug-like properties. The models were built from docking conformers and evaluated by combined survival scores of active and inactive of the models. And then two best models (APRRR215 and AHPRRR104) were chosen by predictive power among their 3D-QSAR models generated from Top10 among five/six-featured models. In the view of screening speed and performance, the pharmacophore models were efficient at finding 2D-novel and 3D-similar scaffolds with known D3R antagonists. Our docking model, as a second predictor, could compensate the limited accuracy of fitness score or predicted pKi through the data combination of the scored values. Moreover, CNS-like property was used as an additional criterion with the two models for choosing promising hit compounds. In conclusion, our current work shows that (1) efficient screening of a large compound database (more than 3 million) is possible; and (2) the interpretation of models is well matched in pharmacophore features and docking poses.

## Figures and Tables

**Table 1:** Twenty-five selective D3R-antagonists used to build the pharmacophore model [14].

Compound	Structure	pKi (D3/D2 Selectivity Ratio)
**6**	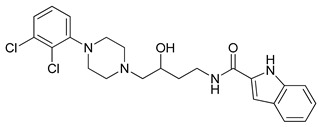	2.73 (397)
**14**	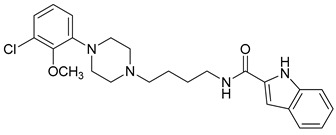	3.41 (135)
**15**	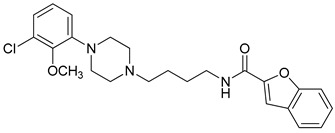	3.04 (80)
**16**	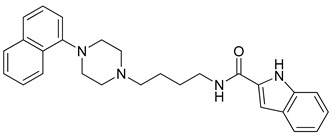	3.93 (109)
**17**	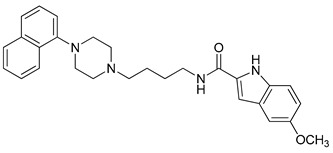	3.76 (80)
**18**	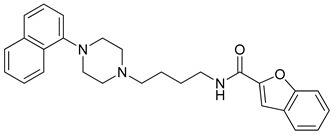	3.61 (61)
**19**	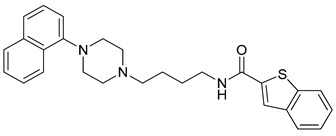	3.89 (65)
**20**	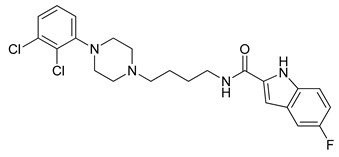	3.55 (131)
**21**	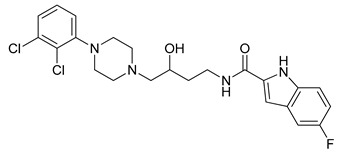	2.74 (182)
**22**	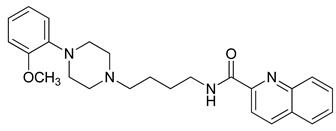	2.46 (19)
**23**	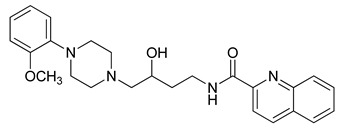	1.01 (9.5)
**24**	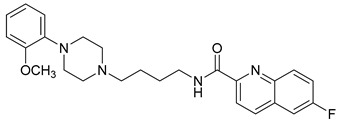	2.60 (27)
**25**	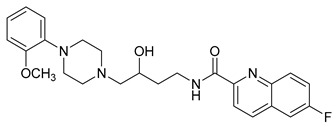	1.27 (19)
**26**	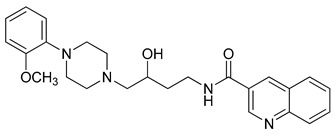	2.67 (16)
**27**	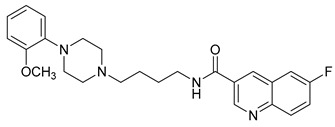	1.35 (15)
**28**	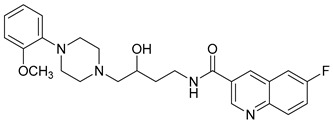	1.28 (13)
**29**	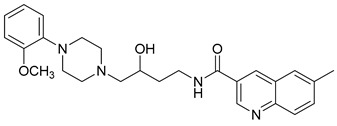	1.16 (11)
**30**	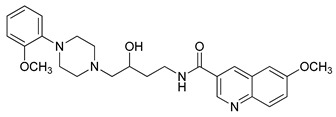	1.42 (27)
**31**	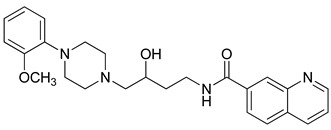	1.42 (27)
**32**	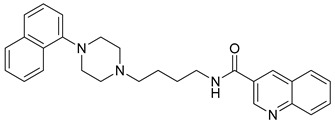	3.45 (45)
**33**	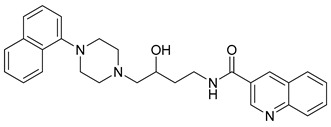	2.44 (71)
**34**	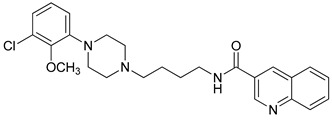	2.70 (22)
**35**	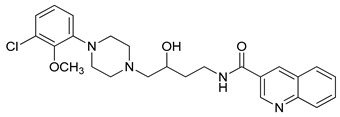	1.50 (28)
**36**	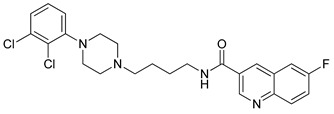	3.24 (69)
**37**	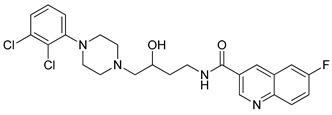	2.09 (103)

**Table 2:** Means of statistic variations of QSAR models.

ID	SD	R-squared	F	Stability	RMSE	Q-squared	Pearson-R
APRRR215	0.436	0.8014	133.1	0.5826	0.4819	0.7347	0.885
HPRRR1639	0.38	0.8491	185.7	0.7004	0.5094	0.7036	0.8815
HPRRR1660	0.3378	0.8807	243.7	0.6467	0.5307	0.6783	0.8809
HPRRR133	0.506	0.7324	90.3	0.8561	0.5593	0.6426	0.8369
ADPRR111	0.4345	0.8027	134.3	0.3582	0.5645	0.6361	0.8363
AHPRRR.104	0.4177	0.8177	148	0.6505	0.4973	0.7175	0.8591
ADHPRR.82	0.4748	0.7644	107.1	0.5688	0.5748	0.6226	0.8293
DHRRR.673	0.3455	0.8753	231.6	0.555	0.581	0.6143	0.8167
AHPRRR.551	0.4102	0.8241	154.6	0.4573	0.5861	0.6076	0.8147
AHPRRR.554	0.4102	0.8241	154.6	0.4573	0.5861	0.6076	0.8147

**Table 3:** Structure, estimated efficacy and ADME properties of selected hit compounds.

No	Structure	D3R ^a^(%)	D2R ^a^(%)	Ro5 ^b^	Ro3 ^c^	CNS ^d^	LE ^e^	G-score ^f^	Pred. Actg ^g^
1	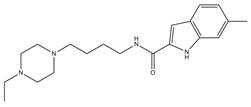	29.3	4	0	0	1	−0.30	−7.56	3.11, 3.11
2	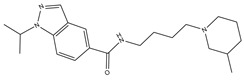	30.8	20.6	0	0	1	−0.31	−8.02	2.68, 2.58
3	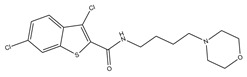	15.8	11.4	0	0	2	−0.31	−7.34	2.83, 2.87
4	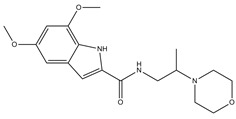	13.5	17.6	0	0	1	−0.30	−7.57	2.82, 2.65
5	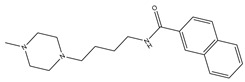	9.1	−4.4	0	0	2	−0.33	−7.87	2.98, 2.86
6	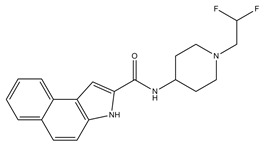	NT ^h^	NT ^h^	0	0	1	−0.32	−8.32	2.54, 2.61
7	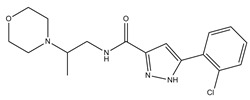	−5.4	−0.1	0	0	1	−0.34	−8.04	2.75, 2.84
8	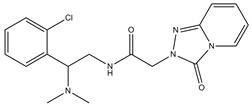	11.9	3.8	0	0	1	−0.32	−8.29	2.31, 2.19
9	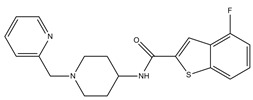	54.1	97.2	0	0	1	−0.30	−7.87	2.55, 2.53
10	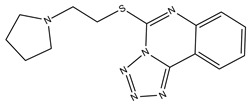	14.8	15.7	0	0	1	−0.34	−7.08	2.40, 2.40

^a^ Percent efficacy of antagonists was measured at 10 uM in GPCR biosensor assay. For antagonist assay, data was normalized to the maximal and minimal response observed in the presence of dopamine (control ligand) and vehicle. The following EC80 concentrations were used for D3R & D2R arrestin assay (D3R: 0.072 μM Dopamine, D2R: 0.2 μM); ^b^ Rule of five; ^c^ Rule of three; ^d^ CNS-likeness; ^e^ Ligand efficiency; ^f^ The Docking score was acquired through the abstraction of Epik penalty from Glide score; ^g^ Predicted activity under two models; ^h^ NT = not tested.

## Supplementary Materials

The supplementary materials are available online.
